# Overview and comparison of the clinical scores in hidradenitis suppurativa: A real-life clinical data

**DOI:** 10.3389/fmed.2023.1145152

**Published:** 2023-04-17

**Authors:** Mathieu Daoud, Mariano Suppa, Farida Benhadou, Mathilde Daxhelet, Hassane Njimi, Jonathan White, Gregor Jemec, Véronique del Marmol

**Affiliations:** ^1^Department of Dermatology, Hôpital Erasme, Université Libre de Bruxelles (ULB), Brussels, Belgium; ^2^Department of Dermatology, Zealand University Hospital, Roskilde, Denmark; ^3^Health Sciences Faculty, University of Copenhagen, København, Denmark

**Keywords:** hidradenitis suppurativa, acne inversa, scoring, severity, phenotypes, personalized medicine

## Abstract

**Introduction:**

Partly due to its clinical heterogeneity, hidradenitis suppurativa (HS) is difficult to score accurately; illustrated by the large number of disease scores. In 2016, a systematic review by Ingram et al. reported the use of about thirty scores, and since then, this number has increased further. Our aim is twofold: to provide a succinct but detailed narrative review of the scores used to date, and to compare these scores with each other for individual patients.

**Materials and methods:**

The review of the literature was done among articles in English and French, on Google, Google scholar, Pubmed, ScienceDirect and Cochrane. To illustrate the differences between scores, data from some Belgian patients included in the European Registry for HS were selected. A first series of patients compares the severity of the following scores: Hurley, Hurley Staging refined, three versions of Sartorius score (2003, 2007, 2009), Hidradenitis Suppurativa Physician Global Assessment (HS-PGA), International Hidradenitis Suppurativa Severity Scoring System (IHS4), Severity Assessment of Hidradenitis Suppurativa (SAHS), Hidradenitis Suppurativa Severity Index (HSSI), Acne Inversa Severity Index (AISI), the Static Metascore, and one score that is not specific to HS: Dermatology Life Quality Index (DLQI). A second set of patients illustrates how some scores change over time and with treatment: Hurley, Hurley Staging refined, Sartorius 2003, Sartorius 2007, HS-PGA, IHS4, SAHS, AISI, Hidradenitis Suppurativa Clinical Response (HiSCR), the very new iHS4-55, the Dynamic Metascore, and DLQI.

**Results:**

Nineteen scores are detailed in this overview. We illustrate that for some patients, the scores do not predictably and consistently correlate with each other, either in an evaluation of the severity at a time-point t, or in the evaluation of the response to a treatment. Some patients in this cohort may be considered responders according to some scores, but non-responders according to others. The clinical heterogeneity of the disease, represented by its many phenotypes, seems partly to explain this difference.

**Conclusion:**

These examples illustrate how the choice of a score can lead to different interpretations of the response to a treatment, or even potentially change the results of a randomized clinical trial.

## Introduction

Hidradenitis suppurativa (HS), ICD-11 code ED92.0, is a chronic, inflammatory, recurrent, debilitating skin disease that usually presents after puberty with painful, deep-seated, inflamed lesions in the apocrine gland-bearing areas of the body, most commonly the axillary, inguinal and anogenital regions (1).

The assessment (e.g., a numerical score) of the severity of HS is complex and has remained controversial for more than a century, from the time Aristide Verneuil first described the disease in 1854, (2) until 1989, when Hurley defined the eponymous score that is still most frequently used (3). In recent years, especially since the development of new targeted therapies, the number of severity scores continues to increase year by year. Some of them are based on objective criteria, while others leave room for subjective evaluation of the patient’s pain or other complaints. Some are complex and time-consuming, while other scores are simplified and easy to use in a clinical setting. The scores can be roughly distinguished between staging systems (pure classification into categories) and outcome measurements instruments (OMI), that aims to measure changes in health status (4).

On closer inspection, staging systems and OMI are always comprised of a set of items combined into a formula, which gives the final score (or category). These items can be elementary lesions (nodules, abscesses, fistulae, cords, follicular papules/pustules, comedos, multiple pyogenic granulomas), (5) quality of life data, or other objective/subjective measures. Many scores share the same criteria, which sometimes allows several scores to be calculated with the same clinical data (6).

### Objective

The first objective of this work is to provide an overview/narrative review of the scoring systems used in hidradenitis suppurativa.

The second objective is to compare the scores with each other, highlight their discrepancies and discuss the potentially major impact of the choice of one or the other score in a clinical study.

## Materials and methods

The review of the literature was performed using articles in English and French, on Google, Google scholar, Pubmed, ScienceDirect and Cochrane. The term “hidradenitis suppurativa” and its Mesh synonyms were used, as well as translations into French according to the GDT (*Grand Dictionnaire Terminologique*). The terms “severity,” “score,” “scoring,” “outcome,” “evaluation,” and “assessment” were also used as keywords.

To meet our second objective, to illustrate the possible contradiction between some scores, we selected Belgian patients included in the European Registry for HS (ERHS), (7, 8) with fully completed data to allow calculation of all evaluable scores.

For a first series of patients, we compared 12 scores estimating their severity, at a given time: Hurley, Hurley Staging refined, three versions of Sartorius score (2003, 2007, 2009), Hidradenitis Suppurativa Physician Global Assessment (HS-PGA), International Hidradenitis Suppurativa Severity Scoring System (IHS4), Severity Assessment of Hidradenitis Suppurativa (SAHS), Hidradenitis Suppurativa Severity Index (HSSI), Acne Inversa Severity Index (AISI), the Static Metascore and the Dermatology Life Quality Index (DLQI).

For a second set of patients, we compared the evolution of 12 scores from the moment the patient starts adalimumab (baseline) to a follow-up around 12 weeks (8 to 15 weeks). The scores compared are the following: Hurley, Hurley Staging refined, Sartorius 2003, Sartorius 2007, HS-PGA, IHS4, SAHS, AISI, Hidradenitis Suppurativa Clinical Response (HiSCR), iHS4-55, the Dynamic Metascore, and the DLQI. For the calculation of the dynamic metascore, the evolution of the DLQI was made according to the cut-offs described by Hongbo et al. (9).

Some measures have been taken to minimize any bias and calculation error, as follows.

Firstly, the ERHS works by collecting the items needed for the scores, (6) so the investigator does not have to calculate the score in front of the patient. This not only avoids calculation errors, but also fixes the counting of lesions between scores (e.g., the number of draining fistulas in the IHS4 or in the SAHS is the same, the investigator only had to count them once).

Secondly, for the second cohort of patients, we ensured that it was always the same investigator reviewing the patient between the baseline visit and follow-up. Indeed, it was shown that for most scores the inter-rater reliability was very poor, while the intra-rater reliability was very good or even excellent (4, 10).

## Results

### Results for the first objective: Scores overview

**FIGURE 1:**
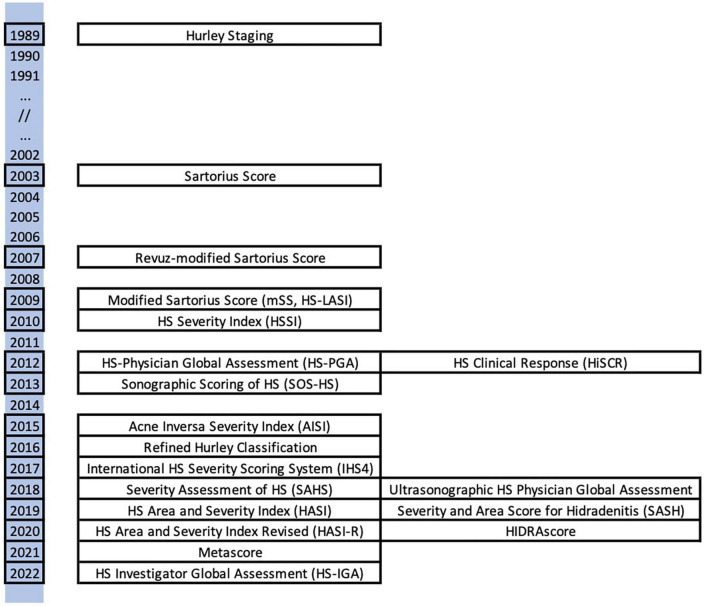
Illustration of the scores detailed inthis article, spread over a timeline.

Figure 1 represents the large number of scores that have been created to quantify HS and shows that the interest in finding an ideal score has increased in recent years. The scores illustrated in this figure are all detailed below.

### Hurley staging (1989)

Hurley’s score (3) is the first, the best known, the most frequently used and cited one, and certainly the preferred tool in clinical practice, (11) its influence being probably much greater than expected by Hurley himself (12).

A simple and recent description of the three Hurley stages was given in the review by Saunte et al. (13).

- Stage I: transient non-scarring inflammatory lesions.
- Stage II: separate lesions consisting of recurrent abscesses with tunnel formation and scarring, and single or multiple lesions separated by normal-looking skin.
- Stage III: coalescent lesions with tunnel formation, scarring, and inflammation.

In its original description, stage III was defined as “diffuse or near-diffuse involvement of multiple interconnected lesions across an entire area.” This concept of “entire area” has been clarified in 2012 by the Hidradenitis Suppurativa Foundation (as described by J. Revuz):(14) the whole or almost the whole anatomical region, except for the buttocks, where it must correspond to 1% of the body surface area (BSA). This view is the most commonly used in practice, and also corresponds to the description of Hurley 3 in the Refined Hurley score, described below (15). It is worth remembering that this classification applies to a region and not to a patient. It is generally accepted that if a patient has multiple affected areas at different Hurley stages, the most severe stage will be the one that defines the patient’s category.

The Hurley score is more consistent with the assessment of the damage caused by the disease than its evolution (14), being then too static to assess treatment response. It essentially records the peak of scarring “frozen in time.” Additionally, this score does not take into account subjective elements (patient-reported outcomes, PROs) at all (16).

### Sartorius score and its modifications (2003–2007–2009)

Sartorius et al. proposed a new scoring system in 2003, (17–19) referred to as the “Sartorius score,” or “Original Sartorius Score.”

This score was initially described as follows: (18)

1. *Anatomical region involved* (axilla, groin, gluteal or other region or inframammary region left and / or right: three points per region involved).
2. *Number and scores of lesions* (abscesses, nodules, fistulas, scars: points per lesion of all regions involved: nodules 2; fistulas 4; scars 1; others 1).
3. *The longest distance between two relevant lesions*, i.e., nodules and fistulas, in each region, or size if only one lesion (<5 cm, 2; <10 cm, 4; >10 cm, 8).
4. *Are all lesions clearly separated by normal skin?* In each region (yes 0 / no 6).

**FIGURE 2:**
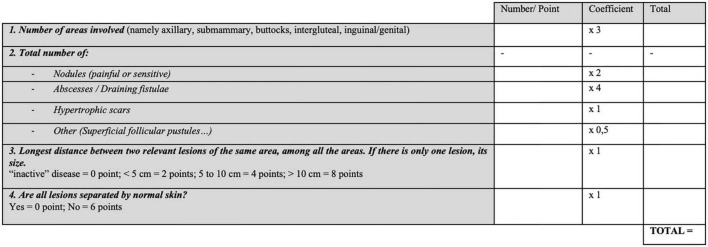
Revuz-Modified Sartorius score 2007 (19).

This score was edited in 2007 by Jean Revuz in an article written in French, creating a new score known as the “*Score de Sartorius modifié*,” in English “modified Sartorius Score,” illustrated in Figure 2 (19). The modifications are:

- five typical regions instead of four. Indeed, he distinguishes the gluteal (convex and frictional location) from the inter-gluteal location, where lesions are often very different.
- a lower coefficient for folliculitis-type lesions, as these lesions have much less impact on daily life than the typical inflammatory lesions.
- the possibility of a “0 point” rating if the distance between two lesions is zero, (inactive disease).

In addition, Jean Revuz provides precisions, a “user manual,” where he specifies that atypical locations, such as the thorax or the retro-auricular sulcus, do not count as additional locations. Regarding the count of lesions in this score, we should only count painful or sensitive nodules after palpation. Moreover, soft scars and post-operative scars do not count.

**FIGURE 3:**
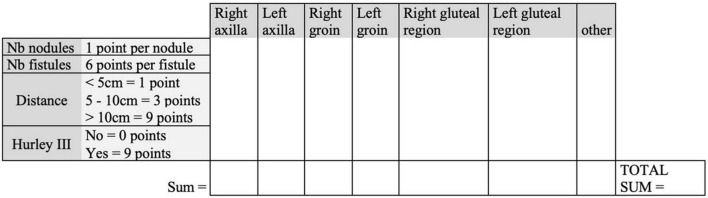
Modified Sartorius score 2009 (17). Nb, number of; Distance, maximal distance between two lesions in the concerned area.

In a study on risk factors in HS published in 2009, Sartorius redefined her own score (17), known as the “Modified Sartorius Score” (mSS), the “Hidradenitis Suppurativa Score,” or sometimes HS-LASI (Hidradenitis Suppurativa Lesion, Area and Severity Index) (20). A major source of confusion, particularly for Francophone readers, is that these changes are not the same as those made by Revuz in 2007. The modified Sartorius score of 2009 brings very different precisions, starting with the fact that it is calculated exclusively region by region. The largest distance between two lesions in the same region is therefore added for each region, as well as the number of regions affected by Hurley III. The Modified Sartorius score has a good correlation with the Hurley score, and a fair correlation with the DLQI (17). It is calculated as follows (total sum of points):

- 3 points per region involved, among those present in Figure 3.

- 1 point per number of nodules, six points per number of fistulae.
- For each region, the longest distance between two lesions is calculated: this item will be worth 1 point if less than 5 cm, 3 points between 5 and 10 cm, and 9 points if greater than 10 cm.
- Each Hurley III area will be worth nine points, the Hurley I and II are always worth 0 points.

Many other variants of the Sartorius scores exist, but are rarely cited, e.g., in 2009 by Tierney et al. (21) and in 2011 by Highton et al. (22).

In comparison to Hurley staging, the mSS is much more dynamic (14) and is able to reflect changes in severity between two visits. One of its drawbacks is that it is based solely on objective items, determined during the physical examination of the patient. The authors themselves have suggested supplementing it with two other data: the soreness of the most symptomatic lesion (VAS of pain) and the number of boils in the month preceding the visit (17). Interestingly, these same two items are incorporated into a score created a few years later, and described below, the Severity Assessment of Hidradenitis Suppurativa (SAHS) (23). Unfortunately, in all versions, these scores are time-consuming to calculate, especially in patients with extensive involvement (17).

### Hidradenitis suppurativa severity index (HSSI) (2010)

**FIGURE 4:**
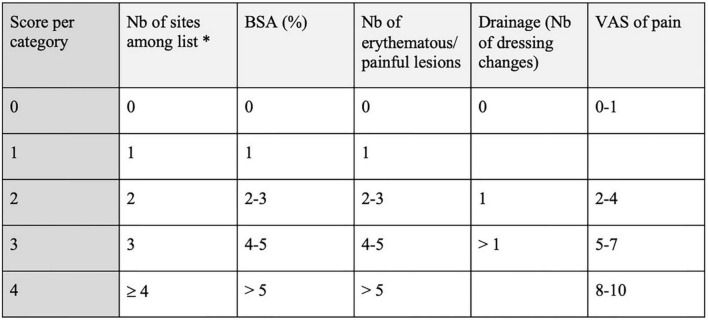
Hidradenitis Suppurativa severity index (HSSI) (24). *Sites: left armpit, right armpit, left side of chest, right side of chest, left groin, right groin, perianal, and sacral, perineal. Nb, number; BSA, body surface area; VAS, visual analog scale.

The final version of this score was described in 2010 (24), in a study on infliximab. It is composed of five items: the number of lesions, the body surface area (BSA), the number of erythematous and/or painful lesions, the number of dressing changes during work and leisure hours (item “drainage”), and a pain-scale from 0 to 10. Each of these items is a value that is referenced to an index from 0 to 4, as shown in Figure 4. The HSSI is the sum of the five indices.

The maximum value of this score is not 20 but 19 points, because the item “drainage” has a maximum index of 3. The severity of the disease is considered as “mild” if the score is ≤7, “moderate” if 8–12, and “severe” if≥13. An older version of this score did not include the number of lesions (25).

This HS-specific score is actually the only one before 2019 that can be calculated without having to distinguish between the different elementary lesions. This feature makes it an easy and quick score to use but is less detailed than the modified Sartorius score, and is not validated.

### HS physician’s global assessment scale (HS-PGA, PGA) (2012)

**FIGURE 5:**
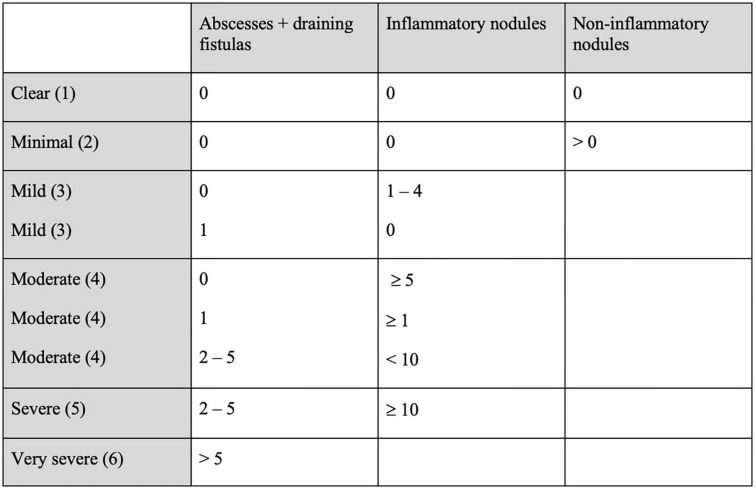
Hidradenitis Suppurativa Physician’s Global Assessment (HS-PGA, PGA) (26).

The HS-PGA (26) is a score that classifies patients into six categories of severity, according to only four items found on physical examination: the number of abscesses, the number of draining fistulas, the number of inflammatory nodules and the presence/absence of non-inflammatory nodules. It is therefore quick and easy to use (see Figure 5) (14), but very dependent on the clinician’s ability to distinguish the HS elementary lesions.

### Hidradenitis suppurativa clinical score (HiSCR) (2012)

The HiSCR (27) is an outcome measurement instrument, based on the same items as the HS-PGA. This score does not give the severity of a patient at a specific time, but the improvement (or not) of the patient between two time-points, e.g., under the effect of a treatment. It will therefore classify patients in two categories:

- Responders to treatment: at least a 50% reduction from baseline in the abscesses and inflammatory nodules count, with no increase of abscesses or draining fistulae count.
- Non-responders to treatment: in any other case.

Its twofold nature makes this score ideal for clinical studies, and was used for the first time as a primary endpoint in the PIONEER studies (28). A variant of this score, where a reduction of 25 instead of 50% in total number of inflammatory nodules and abscesses is required, is recommended in clinical practice according to the guidelines of the British Association of Dermatologists (29).

Despite being quick, easy to use, widely used in research and practice (11), and despite its verified convergent validity (30), this score has some drawbacks. Firstly, this score was created, tested and validated for patients with baseline total abscesses and inflammatory nodules count of at least three, and draining fistulae count of twenty or fewer (27). Also, since a new draining fistula or abscess is sufficient to classify a patient as a non-responder, this score may be considered too rigid (31). Finally, as for the HS-PGA, this score is very dependent on the clinician’s ability to distinguish the HS elementary lesions.

### Acne inversa severity index (AISI) (2015)

**FIGURE 6:**
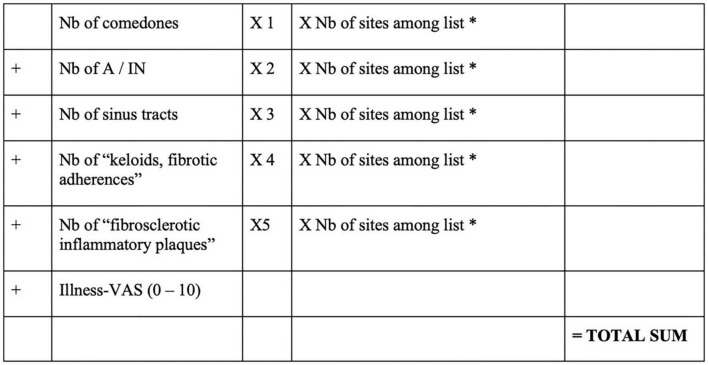
Acne Inversa Severity Index (AISI) (32). *Sites: face, scalp, right axilla, left axilla, right breast, left breast, trunk, right arm, left arm, right groin, left groin, right gluteus, left gluteus, genital area, perianal, right leg, and left leg. Nb, number; A, abscesses; IN, inflammatory nodules; VAS, visual analog scale.

The AISI, (32) detailed in Figure 6, is the first score to consider a patient-reported outcome: the “illness-VAS” (visual analog scale of pain, discomfort, and disability). This score is more complex and takes into account types of lesions that are not described as typical elementary lesions of the disease. Daxhelet et al. (5) furthermore, it could correlate closely with Hurley and DLQI, but its design was created using data from only 46 patients, which may well be insufficient to allow further comment (33). The patient is considered mild if the score is below 10, moderate from 10 to 18, and severe if the score is above 18.

### The refined hurley classification (2016)

**FIGURE 7:**
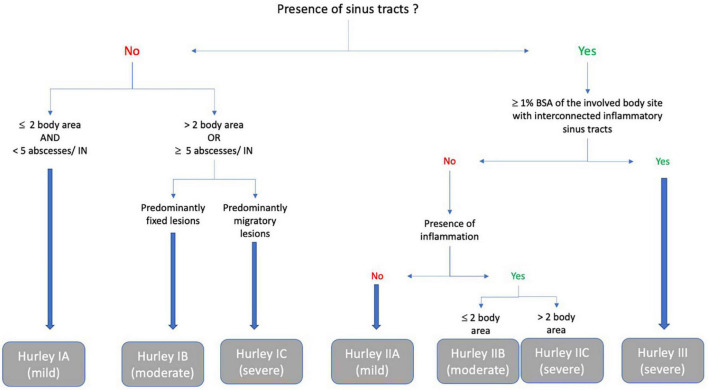
Refined Hurley (15).

This three-step algorithm classifies patients into seven categories, modeled on the Hurley classification (15). Hurley stages I and II are each subdivided into stages A, B, and C (mild, moderate, and severe) according to the degree of inflammation and the extensiveness. Stage III remains unchanged, reflecting severe disease (see Figure 7).

The aim of this score is not to measure the therapeutic effectiveness of a treatment, but mainly to guide treatment decisions (12). For example, some Hurley I patients become severe and would benefit more from anti-TNF-alpha treatment than some mild Hurley II patients.

Stage I and II subcategories A, B, and C have been validated in comparison to the IHS4 and DLQI (34).

### International hidradenitis suppurativa severity scoring system (IHS4) (2017)

A two-round Delphi voting process was conducted among the members of the European Hidradenitis Suppurativa Foundation (EHSF) to create this easy-to-use formula, (33) that shares the same items as the HiSCR: number of nodules, number of abscesses and number of draining fistulae:


I⁢H⁢S⁢4=N⁢o⁢d⁢u⁢l⁢e⁢s+2⁢(A⁢b⁢s⁢c⁢e⁢s⁢s⁢e⁢s)+4⁢(D⁢r⁢a⁢i⁢n⁢i⁢n⁢g⁢f⁢i⁢s⁢t⁢u⁢l⁢a⁢e)

ISH4 is therefore a whole number, reflecting mild disease (0–3), moderate (4–10) and severe ≥11. This score correlates well with Hurley, PGA and mSS, but less well with DLQI. As with all the scores described so far, this score lacks patient-reported outcomes.

In addition to indicating the severity of the disease, this score can be used in a twofold way, like the HiSCR. This is the IHS4-55 (35) developed and validated in 2022, and is used as follows: patients with at least a 55% reduction in their IHS4 between two visits are responders, the others are non-responders. This 55% threshold was determined and validated using de-identified data from the PIONEER studies (28). IHS4-55 was also shown to be discriminating in a prospective study evaluating the efficacy and tolerance of recommended antibiotics in HS (36). In contrast to the HiSCR, this new score also applies to patients whose initial work-up includes fewer than 3 inflammatory lesions, or more than 20 draining fistulae.

### Severity assessment of hidradenitis suppurativa (SAHS) (2018)

**FIGURE 8:**
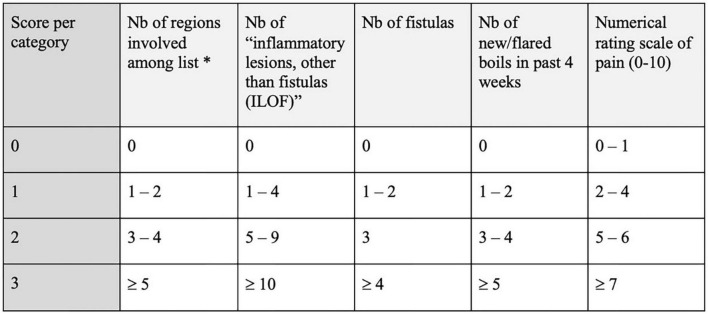
Severity Assessment of Hidradenitis Suppurativa (SAHS) (23). *Sites: axilla left, axilla right, submammary left, submammary right, intermammary/chest, abdominal, mons pubis, groin left, groin right, genital, perianal, gluteal left, gluteal right, and others. Nb, number.

The SAHS, (23) detailed in Figure 8, is a value ranging from 0 to 15, depending on 5 items: the number of locations, fistulas, inflammatory lesions that are not fistulas, flare-ups (in the last 4 weeks), and a numerical pain scale. The final score may reflect mild (if below 4), moderate (5–8) or severe (above 9) disease.

This score, which has been validated in comparison with the Hurley and the mSS, has advantages that have been pointed out by many experts (37, 38).

Its main novelty is that it offers a combination of clinical items and subjective items (patient-reported outcomes). The item “Number of new or flared existing boils in past 4 weeks” is a welcome additional criterion since HS is characterized by periods of relapses and remissions. The insertion of an “ILOF” item (inflammatory or painful lesions that are not fistulas) rather than a count of separate nodules and abscesses makes it relatively easy to calculate the score.

### Hidradenitis suppurativa area and severity index revised (HASI-R) (2020)

Hidradenitis suppurativa area and severity index revised (39) was created by combining two other scores, published in 2019: the Severity and Area Score for Hidradenitis (SASH) (40), and the Hidradenitis Suppurativa Area and Severity Index (HASI) (41).

These scores were developed by the HIdradenitis SuppuraTiva cORre outcome set International Collaboration (HISTORIC), whose objective is to create a list of outcomes to be measured in any clinical trial on HS (Core Outcome Set) (42–44). They aim to eliminate all biases related to lesion counting (31), which is sometimes complicated in HS, especially in patients with a large number of lesions. New items, inspired by the Psoriasis Area Severity Index (PASI), replace the HS elementary lesions: discoloration (related to inflammation), induration (related to inflammation), extent of fistula and ulceration. These items are multiplied by a coefficient reflecting the body surface area (BSA) for 10 body sites.

Although this score is intended to be simpler to use, avoiding the often inaccurate counting of the individual lesions, the assessment of inflammation-related color can be difficult on darker skin. The study involved fifteen patients with phototypes ranging from I-IV. It can also be difficult to distinguish between inflammation-related induration and the elevation of certain scars (31). However, in a cohort of 20 physicians evaluating 15 patients, the HASI-R shows excellent intra-rater reliability, and moderate inter-rater reliability, but better than five other scores based on lesion counts (39).

### HIDRAscore (2020)

The HIDRAscore (45) is calculated on the basis of five items: the number of inflammatory nodules, abscesses and draining fistulas, the presence or absence of subumbilical lesions, and the HIDRAdisk, a quality-of-life index specific to HS (46). The introduction of the item “presence of subumbilical lesions” is justified by the fact that these lesions are more severe, have a greater impact on quality of life, and are often more difficult to treat (47).

### Metascore (2021)

The metascore (48) is a combination of the other scores mentioned, and is designed to be applied at the cohort (group) level, not at the patient level. Provided that the scores that the investigator wishes to evaluate are known, a cohort of patients could be classified from the most severely affected to the least severely affected patient (static metascore). A cohort could also be classified according to the response to a treatment, from the best responder to the worst responder (dynamic metascore).

### Hidradenitis suppurativa investigator global assessment (HS-IGA) (2022)

Like the HASI-R, the HS-IGA is a score developed with the help of the HISTORIC initiative, by HS experts, patient research partners and outcome measure development experts (49). Once again, this score can be calculated without having to distinguish between elementary lesions (apart from scars). Indeed, abscesses, nodules (inflammatory or not) and fistulas (draining or not) all have the same weighting in the final calculation of the score and therefore do not have to be distinguished. Another major simplification is that there are only two possible locations: “upper body” and “lower body,” the delimitation of is at the umbilicus.

The patient is considered a treatment responder if the HS-IGA decreases by two points or more.

### Other clinical assessment methods

As outlined by John Ingram in 2016 (50), the number of HS assessment tools is very large, including scores that have been used in only one study or none at all.

This is the case with the Physician Global Assessment of Hidradenitis Suppurativa Lesions (51), used in a study on Etanercept in HS. This score focuses on the one hand on a target lesion, and on the other hand on an overall assessment.

Another very simple score combining physical examination data (the number of inflammatory lesions, ILs) with the DLQI has been proposed by Sabat et al. (37, 52) distinguishing 3 categories: mild (1–4 ILs and 0–10 DLQI), moderate (5–10 ILs or 11-20 DLQI) and severe (>10 ILs or 21–30 DLQI).

### Imaging scores

Ultrasound is of interest in HS, particularly in assessing its severity. There are mainly two scores: the Sonographic Score of Hidradenitis Suppurativa (SOS-HS) (53) and the Ultrasound-HS-PGA (54). There is a relevant disagreement between clinical scores and ultrasound scores (54), probably because ultrasound detects deeper and potentially less clinically obvious lesions, such as incipient fistulae.

Finally, thermography could have a place in the assessment of the severity of HS, but this method has so far been used more in a diagnostic or therapeutic context (surgical margins) (55, 56).

### Patient-reported outcome measures

Scores evaluating the quality of life of HS patients are numerous and widely used, since HS-related depression is not necessarily correlated with the severity scores mentioned above (57). These include, for example, the previously mentioned DLQI (58), widely known and used because it is well suited to HS as well as other skin conditions (59), or specific HS tools such as HiSQoL (60) or HIDRAdisk (46). All these scores are outside the scope of this article and are discussed in other works (31).

### Results for the second objective: Scores comparison

#### First series of patients: comparison of severity scores at a given time

Five patients with all the items needed to calculate the desired scores were selected. Their scores are all detailed in Table 1. For each of the five patients, the score (number, continuous value) and its interpretation (severity category) are indicated. For scores that do not have severity categories (Sartorius), “N/A” is indicated. For the metascore, simply ranking patients from least to most severe, the two most extreme cases were specified, and the others were indicated as “intermediate.” A color gradient from green (low score, mild impairment) to red (high score, severe impairment) is used to better illustrate the discrepancies between the scores.

**TABLE 1:** Comparison of severity scores at a given time.

Patient Nr	290		447		507		561		567	
	**Value**	**Interpretation**	**Value**	**Interpretation**	**Value**	**Interpretation**	**Value**	**Interpretation**	**Value**	**Interpretation**
DLQI (effect on patient’s life)	5	Small	18	Very large	3	Small	4	Small	29	Extremely large
Hurley	2	Moderate	3	Severe	2	Moderate	2	Moderate	1	Mild
Hurley Refined	IIA	II-Mild	III	III-Severe	IIB	II-Moderate	IIB	II-Moderate	IB	I-Moderate
Sartorius 2003	48	N/A	60	N/A	27	N/A	42	N/A	25	N/A
Sartorius 2007	21	N/A	43.5	N/A	31	N/A	17	N/A	16	N/A
Sartorius 2009	16	N/A	45	N/A	19	N/A	7	N/A	20	N/A
HS-PGA	2	Minimal	4	Moderate	4	Moderate	3	Mild	1	Clear
iHS4	0	Mild	5	Moderate	9	Moderate	2	Mild	0	Mild
SAHS	7	Moderate	9	Severe	7	Moderate	5	Moderate	4	Mild
HSSI	12	Moderate	13	Severe	10	Moderate	5	Mild	5	Mild
AISI	11	Moderate	13	Moderate	9	Mild	7	Mild	4	Mild
Rank in static metascore	3	Intermedate	5	Most severe	4	Intermediate	2	Intermediate	1	Less severe

For each of the five patients, the score (number, continuous value) and its interpretation (severity category) are indicated. For scores that do not have severity categories (Sartorius), “N/A” is indicated. For the metascore, that simply ranks patients from least to most severe, the two most extreme cases were specified, and the others were indicated as “intermediate”. A color gradient from green (low score, mild impairment) to red (high score, severe impairment) is used to better illustrate the discrepancies between the scores. N/A, not applicable; DLQI, dermatology life quality index; HS-PGA, hidradenitis suppurativa physician global assessment; iHS4, international hidradenitis suppurativa severity scoring system; SAHS, severity assessment of hidradenitis suppurativa; HSSI, hidradenitis suppurativa severity index; AISI, acne inversa severity index (AISI).

We can observe that for each patient there is at least one discrepancy between the scores. Patient 567 has a near-maximum DLQI (indicating an extremely large impact on the patient’s life, according to Hongbo et al. categories) (9) while in the HS specific scores his impairment is clear, mild, or moderate.

#### Second series of patients: Comparison of the evolution of scores after circa 12 weeks of adalimumab

Nine patients with all the items needed to calculate the desired scores, and evaluated by the same rater between baseline and follow-up (after 8 and 15 weeks of adalimumab), were selected. Their scores are detailed in Table 2. A color gradient from green (low score, mild impairment) to red (high score, severe impairment) is used to visually contrast the change between the baseline and follow-up visits. Also, their phenotype is given as an indication, according to the Dudink et al. classification (61). Four patients are frictional furuncles type, four are regular type, and one is scarring folliculitis type.

**TABLE 2:** Comparison of the evolution of scores after 8 to 15 weeks of adalimumab.

Patient Nr	106		114		116		481		94		147		183		277		434	
**Phenotype**	**Regular**		**Regular**		**Regular**		**Regular**		**Frictional Furunculoid**		**Frictional Furunculoid**		**Frictional Furunculoid**		**Frictional Furunculoid**		**Scarring Folliculitis**	
**Visit**	**Base-** **line**	**Follow-up**	**Base-** **line**	**Follow-up**	**Base-** **line**	**Follow-up**	**Base-** **line**	**Follow-up**	**Base-** **line**	**Follow-up**	**Base-** **line**	**Follow-up**	**Base-** **line**	**Follow-up**	**Base-** **line**	**Follow-up**	**Base-** **line**	**Follow-up**
DLQI	11	3	14	24	16	8	15	11	13	1	10	4	28	5	13	10	25	6
Hurley	2	2	2	2	2	2	3	2	2	2	2	2	2	2	3	2	2	2
Hurley Refined	IIC	IIC	IIC	IIC	IIC	IIA	III	IIC	IIC	IIA	IIC	IIA	IIB	IIC	III	IIC	IIB	IIA
Sartorius 2003	133	97	43	31	30	11	151	75	73	21	59	39	102	70	63	62	21	18
Sartorius 2007	100	56	43	31	35	7	97	69	79	25	54	34	122	36	59	50	23	15
HS-PGA	6	3	4	3	4	2	6	6	4	1	4	1	6	4	5	4	4	1
IHS4	23	4	10	2	4	0	68	29	20	0	10	0	70	6	22	12	15	0
SAHS	15	6	11	9	8	3	14	14	11	4	8	4	13	8	13	9	10	6
AISI	10	10	10	6	10	4	18	39	9	9	9	9	9	10	5	9	23	14
HiSCR		Responder		Responder		Responder		Non-Responder		Responder		Responder		Responder		Responder		Responder
iHS4-55		Responder		Responder		Responder		Responder		Responder		Responder		Responder		Responder		Responder
Dynamic metascore		5.5		1 (Worst responder)		8		2.5		9 (Best responder)		5.5		5.5		2.5		5.5

For these nine patients, whose phenotype is given as an indication, each score is given at the baseline visit and at follow-up. A color gradient from green (low score, mild impairment) to red (high score, severe impairment) is used to visually contrast the change between the baseline and follow-up visits. DLQI, dermatology life quality index; PGA, hidradenitis suppurativa physician global assessment; iHS4, international hidradenitis suppurativa severity scoring system; SAHS, severity assessment of hidradenitis suppurativa; AISI, acne inversa severity index (AISI); HiSCR, hidradenitis suppurativa clinical response; iHS4-55, international hidradenitis suppurativa severity score system 55.

Here again, we observe that some scores evolve differently than others. For instance, patient 481 is considered an adalimumab responder according to iHS4-55, but not according to HiSCR. For the remaining eight patients, the IHS4 and HiSCR agree. Most scores improve with treatment (i.e., from red to green). Some scores change little, notably the Hurley, which is stable except for two patients, and to a lesser extent the Hurley Staging refined (two patients stable) and the AISI (three patients stable). Sometimes the score shows worsening over time. This is the case for patient 114’s DLQI, patient 481’s AISI, and both patient 183’s Hurley Refined and AISI.

## Discussion

The results show the existence of at least 19 specific HS scores. Two small series of patients illustrate how scores can diverge and sometimes disagree, either in the assessment of a patient’s severity at a given time (Table 1) or in the evaluation of the effectiveness of a treatment (Table 2).

The overview carried out here is not a systematic review and is therefore potentially not complete, but it includes the most cited and discussed scores in cross-sectional articles, as well as in congresses. In 2016, Ingram did a systematic review of treatments used in HS (62), and extracted a list of all outcome measures used in the clinical trials explored (50). In total, 30 outcome measures were described, including a very large number of simple lesion counts. Our overview only included the HS specific scores, not the lesion counts used historically before disease-specific scores were widely used.

It is immediately obvious that the number of scores for HS is very large, while none is unanimously accepted. New scores are still being developed and validated to varying degrees to compensate for the shortcomings of previous scores. Some have become more complex in an attempt to evaluate the disease as comprehensively as possible, while others have sought to simplify the score in order to facilitate its use in clinical practice. Several problems quickly arose: the basic items, i.e., the elementary lesions in most cases, had to be redefined and specified (5). The notion of “flare-up” also had to be clarified: is it a purely subjective notion or should it be quantifiable? (63). The very fact that the disease fluctuates has necessitated a change in the scoring. How can we avoid underestimating severity if the patient is, by chance at the time of the physical examination, between relapses? (64). Some scores have been researched by expert groups, notably the HISTORIC collaboration mentioned above (44), while others have been established by more local teams.

Most studies comparing scores are done experimentally, with patients each being examined by a panel of trained raters, to compare the inter-rater reliability of each score (4, 10). Our study shows real data from a patient registry and compares the scores directly with each other. These data are scarce as the calculation of scores is time-consuming, but this was made possible using ERHS items, and thus the calculation of scores *a posteriori*. This is an advantage, as the risk of miscalculation is minimized.

By observing Table 1, we see the weakness and the limits of certain scores. For instance, patient 290 is categorized as Hurley IIA, therefore having sinus tracts but no inflammatory lesions and consequently has an HS-PGA score considered “minimal,” and an iHS4 score of zero. But looking at HSSI, a score that takes into account body surface area and pain, this patient is only one point away from the “severe” stage. Patient 567 has a DLQI that is completely opposite to the other scores assessed: this indicates an extremely large impact of HS on his quality of life, while the HS-specific scores are clear, mild, or moderate. This highlights the importance of considering scores measuring patients’ quality of life and psychological wellbeing, in addition to severity scores. Indeed, as mentioned in the introduction, HS depression and HS severity are not necessarily well correlated. Sampogna et al. (57) for HS-specific scores, looking at the colors in Table 1, the scores seem to be concordant in very severe (e.g., patient 447) or very mild cases (e.g., patient 567), but are less so in intermediate cases.

Table 2 shows how patient scores changed after circa 12 weeks of adalimumab. Adalimumab is a monoclonal antibody administered subcutaneously at the following dosage: initiation with 160 mg on day zero, 80 mg on week 2 and 40 mg once weekly from week 4. This treatment is particularly effective on inflammatory lesions (abscesses, inflammatory nodules) and drainage, but is not effective alone against non-inflammatory lesions, including hypertrophic scars (65). In Table 2, some examples of discrepancies between the scores are even more striking. For example, patient 114’s DLQI worsened (from 14 to 24) between baseline and follow-up, and is considered the worst responder to adalimumab according to the dynamic metascore. His SAHS is barely modified (from 11 to 9) perhaps reflecting numerous relapses in the preceding 4 weeks. In contrast, the scores which only consider inflammatory lesions were much improved, i.e., considering HS-PGA and iHS4. Hence, patient 114 is considered as a responder to adalimumab according to both HiSCR and iHS4-55.

In a more subtle way, but of great importance, patient 481 presents a very clear improvement in his iHS4 and is considered as “responder” according to iHS4-55. On the other hand, his HS-PGA is unchanged, and this patient is considered as “non-responder” according to the HiSCR. This can be explained, for example, by a very large number of draining fistulas at baseline, which would have improved very well with adalimumab, but with no great change in the number of inflammatory nodules or abscesses. It is the perfect example of the drawbacks of HiSCR, which motivated the creation of the iHS4-55 (and HASI-R). The SHINE study, a phase II randomized clinical trial assessing the efficacy of IFX-1 (vilobelimab) vs. placebo in patients with moderate to severe HS, failed to demonstrate the efficacy of vilobelimab using HiSCR, and in fact suggested a modification of HiSCR (31, 66).

If we focus on HS phenotypes, we can observe that the best responder to adalimumab according to the dynamic metascore is a “frictional furuncles” phenotype patient, and the other three “frictional furuncles” phenotypes patients are also all good responders, in the iHS4-55 as in the HiSCR. We have insufficient evidence to suggest that this phenotype predicts a good response to adalimumab given the small cohort (which is, however, very likely) (65). It demonstrates that this phenotype, rich in inflammatory nodules and abscesses, is very sensitive to changes in scores that inherently take into account these specific lesions. In general, certain phenotypes can be considered more likely to respond according to certain scores. We recommend that the choice of a score, especially in a clinical trial setting, should always be made in consideration of the phenotypes of the patients concerned.

Note that patients 481 and 277 show an improvement in their Hurley score (III to II). This is possible when the reduction of inflammation allows the reappearance of healthy skin between the fistulas and the remaining scars (total involvement is back below 1%). However, a transition from Hurley II to I is impossible without surgery.

The main limitation of this study is the lack of comparison of the HASI-R score and HS-IGA, which are quite recent and for which we still lack experience. Unfortunately, the items required for HiSQOL and some other patient-reported outcomes are not yet included in the ERHS, so could not be included in our analysis.

The number of patients is small because we selected patients whose physical examination included absolutely all the data necessary to calculate all the scores we chose to evaluate. However, some data were only added to the ERHS at a later stage (such as the distance between two lesions for each region, useful for the calculation of the Sartorius 2009, or the notion of migratory lesions necessary for the calculation of the Hurley Refined). Daxhelet et al. (8) in addition, for the dynamic study discussed in Table 2, patients had to have a complete baseline and follow-up visit by the same investigator. This number of patients is not a hindrance for our purposes, as we provide an illustration, not a demonstration.

## Conclusion

Choosing criteria to generate a severity score in HS is always a compromise. Including too much granularity in observation parameters may lead to an impractical tool in the clinic. Including too little detail may result in important fluctuations in the disease activity not being captured. The relative weighting of observer-reported signs against patient-reported symptoms should be carefully negotiated and the choice will essentially depend on which weighting gives the most nuanced information to choose the best treatment options and hence improve the HS patient’s life.

All these components have resulted in a very large number of specific HS scores, which are not necessarily in agreement with each other. The choice of a score can lead to different interpretations of the response to a treatment, or even modify the results of a randomized clinical trial, because according to their phenotypes and other clinical elements, certain patients can be considered as “responders” according to one score, but “non-responders” according to another one. Quality of life scores may differ significantly from severity scores; the psychological aspect and the impact on quality of life is essential to evaluate in addition to the objective evaluation of the severity.

We recommend that in future clinical trials, several measures of disease severity should be used, including objective and patient-reported outcomes, and that the HS phenotypes should always be taken into consideration when deciding on clinical trial protocols.

## Data availability statement

The original contributions presented in this study are included in the article/supplementary material, further inquiries can be directed to the corresponding author.

## Ethics statement

The studies involving human participants were reviewed and approved by Erasme Ethical Committee P2015/071. The patients/participants provided their written informed consent to participate in this study.

## Author contributions

MDao, VM, and GJ conceptualized the project and participated in its methodology. MDao and HN performed the formal analysis. MDao, MS, FB, and MDax actively participated in data collection. MDao wrote the original draft of the manuscript and reviewed by VM, MS, and FB. JW reviewed content and conscientiously. MDao visualized the data. VM provided under the supervision. All authors contributed to the article and approved the submitted version.
